# Antimicrobial activity of supernatants produced by bacteria isolated from Brazilian stingless bee’s larval food

**DOI:** 10.1186/s12866-022-02548-4

**Published:** 2022-05-12

**Authors:** Ana Carolina Costa Santos, Serena Mares Malta, Raquel Cristina Cavalcanti Dantas, Nina Dias Coelho Rocha, Vasco Ariston de Carvalho Azevedo, Carlos Ueira-Vieira

**Affiliations:** 1 Institute of Biotechnology, Federal University of Uberlândia, Acre Street, 2E building, Uberlândia, MG 38405-319 Brazil; 2 Department of Genetics, Ecology and Evolution, Institute of Biological Sciences, Federal University of Minas Gerais, Presidente Antônio Carlos Avenue, 6627, Pampulha, Belo Horizonte, MG 31270-901 Brazil

**Keywords:** Antibiotics, Pathogen, Antibacterial effect, Microorganisms, Multiresistant bacteria, Stingless bees, Larval food

## Abstract

**Background:**

The discovery of new molecules with antimicrobial properties has been a promising approach, mainly when related to substances produced by bacteria. The use of substances produced by bees has evidenced the antimicrobial action in different types of organisms. Thus, the use of bacteria isolated from larval food of stingless bees opens the way for the identification of the new molecules. The effect of supernatants produced by these bacteria was evaluated for their ability to inhibit the growth of bacteria of clinical interest. Furthermore, their effects were evaluated when used in synergy with antibiotics available in the pharmaceutical industry.

**Results:**

A few supernatants showed an inhibitory effect against susceptible and multiresistant strains in the PIC assay and the modulation assay. Emphasizing the inhibitory effect on multidrug-resistant strains, 7 showed an effect on multidrug-resistant *Escherichia coli* (APEC), *Klebsiella pneumoniae* carbapenemase (KPC), multidrug-resistant *Pseudomonas aeruginosa*, and multidrug-resistant *Staphylococcus aureus* (MRSA) in the PIC assay. Of the supernatants analyzed, some presented synergism for more than one species of multidrug-resistant bacteria. Nine had a synergistic effect with ampicillin on *E. coli* (APEC) or *S. aureus* (MRSA), 5 with penicillin G on *E. coli* (APEC) or KPC, and 3 with vancomycin on KPC.

**Conclusion:**

In summary, the results indicate that supernatants produced from microorganisms can synthesize different classes of molecules with potent antibiotic activity against multiresistant bacteria. Thus, suggesting the use of these microorganisms for use clinical tests to isolate the molecules produced and their potential for use.

## Background

Faced with the global concern with public health, which has been facing difficulties in combating resistant pathogens, the search for new drugs with antimicrobial function becomes an emergency. Indiscriminate use of antibiotics results in the selection of resistance to most commercially available antimicrobials [1–3]. Among the classic pathogens, some have a high capacity for the acquisition and dissemination of resistance genes, becoming a public health problem worldwide [4–6]. In this context, bacteria such as *Staphylococcus aureus*, *Escherichia coli*, *Pseudomonas aeruginosa*, and *Klebsiella pneumoniae* stand out, which present multidrug resistance genotypes [2, 4–7].

The antimicrobial effect of products generated by stingless bees has contributed to discovery of biomolecules capable of inhibiting the growth of microorganisms. The pollen of *Melipona compressipes manaosensis* [8], the geopropolis extract of the bee *Melipona quadrifasciata* and *Tetragonisca angustula* [8–10] , as well as the propolis and honey, named by authors as bee bread of *Heterotrigona itama* [11] have shown action against pathogenic microorganisms.

The diversity of microorganisms associated with stingless bee colonies are broad, even as their role in the health and vitality of these organisms [12–16] Microorganisms can contribute to the development of the immune system of bees, assist in food digestion and defend the hive against pathogens [17–19]. *Scaptotigona depilis* bees are related to a mutualistic relationship with fungi of the genus *Zygosaccharomyces*, which influence larval development, survival rate, and differentiation of queens [20–22]. Despite the progress in studies with the microbiota associated with stingless bee colonies, it is still unknown its effective role in the maintenance and development of the colonies. Bacteria associated with stingless bees have biotechnological purposes, such as probiotics, disease biocontrol agents, producers of enzymes, and antimicrobial substances [12, 23].

In this context, studies related to the discovery of new molecules with antimicrobial function obtained from by-products generated by stingless bees may represent an alternative to the global public health problem about antibiotic resistance and treatment of infections caused by multidrug-resistant pathogens. The present investigation aims to evaluate the capability of microorganisms isolated from larval food of stingless bees to generate biomolecules with antimicrobial potential.

## Methods

### Sample collection

This study used 14 bacteria’s isolated from *Melipona quadrifasciata, Melipona scutellaris,* and *Tetragonisca angustula* (Table 1) from the Collection of Microorganisms Isolated from Stingless Bee from the Laboratory of Genetics of Biotechnology of UFU (CoMISBee).

**Table 1:** CoMISBee Bacteria and Bee Code

CoMISBee Code	Bees Larval Food
Mq-ISP-1A	*Melipona quadrifasciata*
Mq-ISP-1B	*Melipona quadrifasciata*
Mq-MCK-7	*Melipona quadrifasciata*
Mq-MRS-9A	*Melipona quadrifasciata*
Mq-MRS-9BI	*Melipona quadrifasciata*
Mq-TSA-12	*Melipona quadrifasciata*
Ta-TSA-14	*Tetragonisca angustula*
Mq-BHI-20	*Melipona quadrifasciata*
Mq-OAT-27	*Melipona quadrifasciata*
Ms-BHI-39A	*Melipona scutellaris*
Ms-BHI-39B	*Melipona scutellaris*
Ta-NUT-45	*Tetragonisca angustula*
Mq-BHI-47	*Melipona quadrifasciata*
Mq-NUT-54B	*Melipona quadrifasciata*

### Supernatant production

**Fig. 1:**
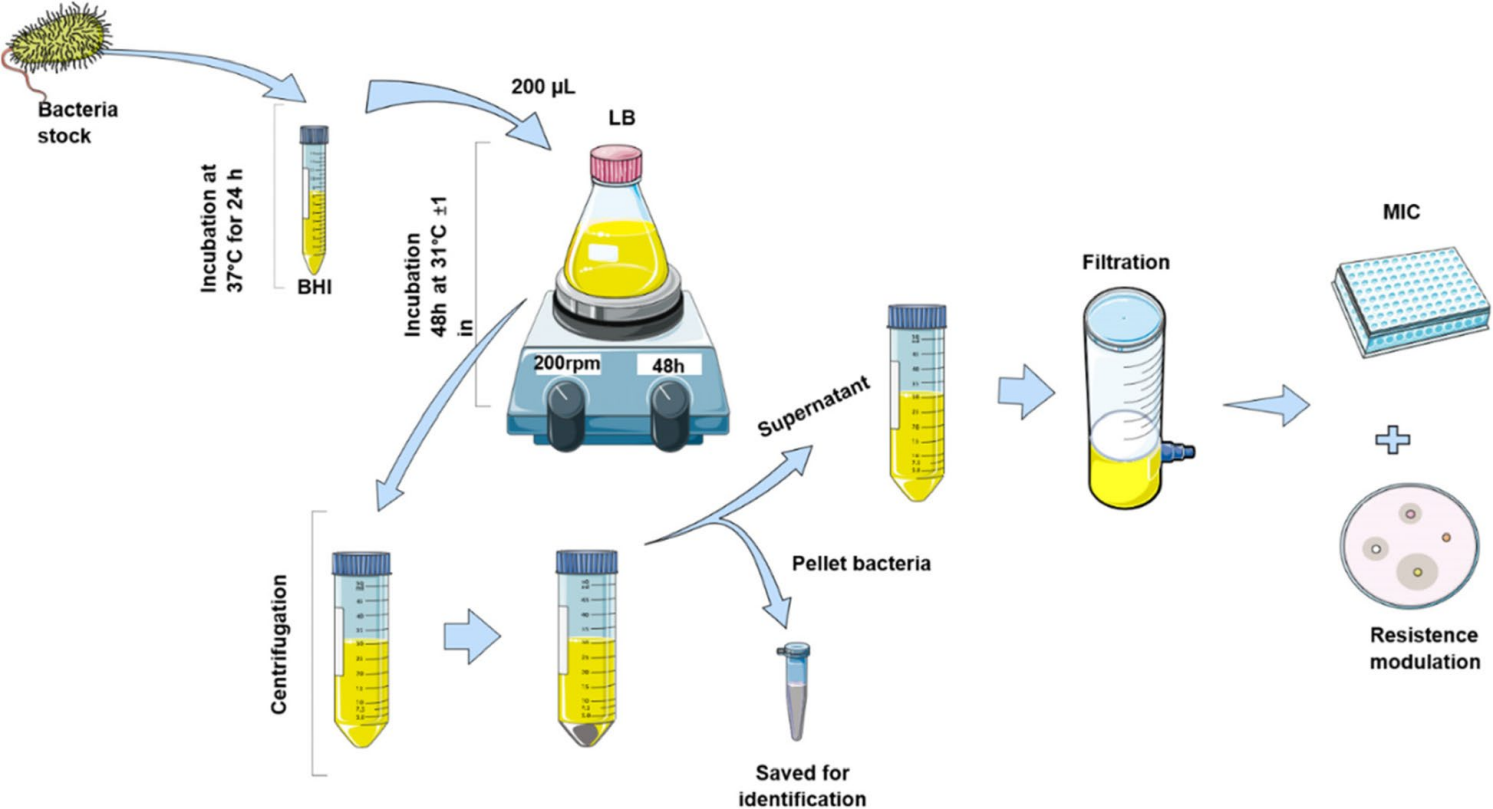
Methodology for supernatant production from microorganisms Collection of Microorganisms Isolated from Stingless Bee (CoMISBee) from the Laboratory of Genetics of UFU. Images of representative metodology from ‘Smart Servier Medical Art’ (https://smart.servier.com/)

For production of supernatant, a bacterial suspension was prepared in 5mL of BHI and incubated at 37ºC for 24 hours. A 200μL rate of the suspension was inoculated in 50mL of LB broth (Luria-Bertani) and incubated at 31ºC±1 for 48h under shaker agitation at 200 rpm. The broth obtained was centrifuged at 10,000 g for 4 minutes for bacterial cell sedimentation [24] and the supernatant was separated from the precipitated and filtered at 0.22 µm (Figure 1). The 14 supernatants was stored in a -20ºC freezer for later use in antimicrobial activity and resistance modulation tests.

### Bacterial identification

For a taxonomic identification at the level of genus and species, the precipitate generated in the centrifugation was inoculated on LB agar plate, and incubated at 37ºC±1 for 24h. An isolated colony of each strain was harvested from the agar using an inoculation loop and inactivated with absolute ethanol. This strain was also submitted to MALDI-TOF mass spectrometry using the MALDI Biotyper version 3 (Bruker Daltonics), according to manufacturer´s suggested settings using automated collected spectra. The biomolecular identification of bacteria were analyzed according to the score values proposed by the manufacturer [25].

### Antimicrobial activity assay

The potential of supernatants to inhibit bacterial growth was evaluated using the Plaque Inhibition Concentration (PIC) method, evaluating for the capacity to kill or inhibit the growth of bacteria with antimicrobial-sensitive and antimicrobial-resistant genotypes. Gram-positive (1) bacteria were used: *Staphylococcus aureus* (sensitive genotype), *Staphylococcus aureus* MRSA (Methicillin-resistant *S. aureus*, multidrug-resistant genotype) and (2) Gram-negative bacteria *Escherichia coli* (ATCC 8739, sensitive genotype), *Escherichia coli* (APEC, multiresistant genotype), Sensitive *Klebsiella pneumoniae*, KPC (Carbapenem-resistant *Klebsiella pneumoniae*, multidrug-resistant genotype) and *Pseudomonas aeruginosa* (sensitive genotype, PAO-1) and *Pseudomonas aeruginosa* (multiresistant genotype). The pathogenic bacteria used were obtained from culture collections or isolated from clinical samples, provided by the Laboratories of Molecular Microbiology (MICROMOL) and Animal Biotechnology Laboratory (LABIO) of the Federal University of Uberlândia.

For the assay, a bacterial suspension was prepared in LB broth at 37ºC±0.5 for 24 hours and diluted to 10^4^ cells⁄mL. Fifty microliters of bacterial suspension was transferred to a 96-well plate containing 50 μL of supernatant to assess whether would reduce microbial growth or kill microorganisms. Wells containing 100 μL of bacteria and LB were used as control of the experiment, positive and negative for bacterial growth, respectively. The plaque was incubated for 24 hours at 37ºC±0.5 and bacterial growth was evaluated in microtiter plate reader at 595 nm at 0; 6; 12 and 24 hours.

### Resistance modulation assay

The supernatants were evaluated for the ability to modulate bacterial resistance when used in synergy with antibiotics that pathogenic bacteria are resistant. Ampicillin (10 Mcg) was tested for strains of *E. coli* (APEC) and *S. aureus* (MRSA), Gentamicin (10 Mcg) for *P. aeruginosa* (multiresistant), Penicillin-G (10 U) for *E. coli* and *K. pneumoniae*, and Vancomycin (30 Mcg) for *K. pneumoniae*. The antimicrobial effect was determined using the modified Kirby-Bauer disk diffusion test (Bauer et al., 1966). The plates were drilled with wells of 6mm inoculated 50 μL of supernatant, along with one antibiotic disc per well. The diameter of the microbial growth halo was evaluated for each supernatant-antibiotic association and compared with the control, containing only the antibiotic disc. The target bacterium was considered sensitive when inhibition zone formation occurred.

### Statistical analysis

Data are expressed as arithmetic means ± standard error of the mean and were analyzed by analysis of variance for two-way (ANOVA), followed by Dunnet post-test using GraphPad Prism software version 8.0.2 (available http://www.graphpad.com/scientific-software/prism/). Statistical significance was considered when *p* < 0.05.

## Results

### Bacterial identification

Of the 14 strains analyzed, six (42.85%) had a probable species identification, two (14.3%) obtained genus identification and six (42.85%) did not obtain reliable identification (Table 2).

**Table 2:** Identification for MALDI-TOF of bacteria isolated

Supernatant Code	Organism (best match)	Score Value	Organism (second match)	Score Value
1A^b^	*Staphylococcus epidermidis*	1.775	not reliable identification	1.672
1B^c^	not reliable identification	1.413	not reliable identification	1.379
7^b^	*Providencia rettgeri*	1.787	not reliable identification	1.691
9A^a^	*Enterococcus faecalis*	2.413	*Enterococcus faecalis*	2.354
9BI^a^	*Providencia rettgeri*	2.065	*Providencia rettgeri*	2.056
12^c^	not reliable identification	1.438	not reliable identification	1.379
14^c^	not reliable identification	1.399	not reliable identification	1.326
20^a^	*Vagococcus fluvialis*	2.272	*Vagococcus fluvialis*	2.247
27^c^	not reliable identification	1.518	not reliable identification	1.485
39A^a^	*Serratia marcescens*	2.041	*Serratia marcescens*	2.01
39B^a^	*Providencia rettgeri*	2.026	*Providencia rettgeri*	1.924
45^c^	not reliable identification	1.335	not reliable identification	1.332
47^c^	not reliable identification	1.56	not reliable identification	1.512
54B^a^	*Providencia rettgeri*	2.05	*Providencia rettgeri*	2.028

^a^secure genus identification, probable species identification, ^b^probable genus identification, ^c^not reliable identification

### Antimicrobial activity assay

**Fig. 2:**
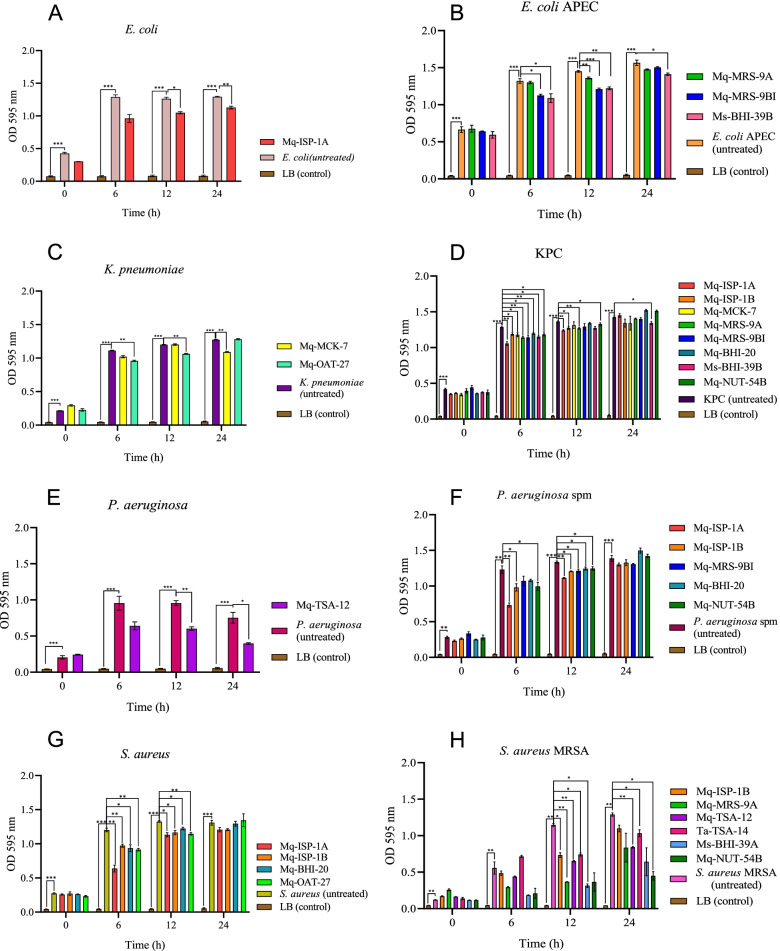
Antimicrobial activity of supernatants produced by microorganisms isolated from stingless bee larval food on sensitive and resistant pathogenic bacteria. The antimicrobial properties of the supernatants were evaluated against strains of sensitive and resistant bacteria. The figure shows only the supernatants that showed a statistically significant difference between the experimental and control groups (bacteria incubated only with LB) in some period of the treatment. Data show the average _ SEM of three independent replicates. *p < 0.05; **p < 0.01; ***p < 0.001

The PIC (Plate Inhibitory Concentration) values of the supernatant for sensitive and resistant pathogenic bacteria are shown in Figure 2. All analyzed supernatants showed an inhibitory effect on more than one bacterium. Sensitive *E. coli*, multidrug-resistant *E. coli*, sensitive *K. pneumoniae*, *carbapenemase K. pneumoniae*, sensitive *S. aureus*, methicillin-resistant *S. aureus*, or multiresistant *P. aeruginosa*.

For sensitive *E. coli*, the supernatant 1A inhibited bacterial growth in 12 and 24 hours of treatment (*p*<0,01) (Figure 2A). The supernatant 39B showed an inhibiting effect on the growth of *E. coli* multidrug-resistant in the 24 hours of treatment (Figure 2B). And the supernatants 9A showed significant effect only 12 hours of treatment (*p*<0,01) and 9BI within 6 (*p*<0,05) and 12 hour of treats (*p*<0,001).

For the species *K. pneumoniae* was observed significant effect by supernatant 07 (*p*<0,01) in 24 hours, (Figure 2C), and 27, in 6 (*p*<0,01) and 12 hours (*p*<0,01). Similarly, several supernatants had an inhibiting effect on KPC in 6 hours, and1A, 1B, 9A and 54 B reduced growth in 12 hoursbut only the 39B (*p*<0,05) supernatant showed a reduction in optical density in 24 hours of bacterial growth (Figure 2D).

Regarding *P. aeruginosa* sensitive, we can highlight the action of the supernatants 12, with an inhibiting effect between the times 6 and 24 hours of growth (Figure 2E). For the multidrug-resistant microorganism, none of the supernatants showed significant effect at the end of growth observed in 24 hours, but the supernatants 1A (*p*<0,01), 1B(*p*<0,05), 9BI (*p*<0,05), 20 (*p*<0,05) and 54B (*p*<0,05) showed significant effect up to 12 hours (figure 2F).

For *S. aureus* sensitive, no antimicrobial effects of the supernatants were observed in 24 hours, and only samples 1A, 1B, 20, and 27 reduced microbial growth to 12 hours, but without significant reduction at the end of 24 hours. For the resistant microorganism (*S. aureus* MRSA), it was possible to verify growth reduction by the growth reducing the effect of supernatants 12, 14 and 54B until the end of the 24 hours of observation. The supernatants 1B (*p*<0,05), 9A (*p*<0,01), 12 (*p*<0,01), 14 (*p*<0,05) and 39A (*p*<0,05), showed a significant effect in the time of 12 hours, although they did not lead to a reduction in bacterial growth in 24 hours of treatment.

### Resistance modulation assay

Sixteen supernatants were evaluated in the resistance modulation assay with multidrug-resistant pathogenic bacteria, which did not present antibiotic inhibition zone. After the addition of the supernatant near the well containing the antibiotic disc, it demonstrated the formation of the inhibition zone for some microorganisms (Table 3).

**Table 3:** Effect of resistance modulation assay using the antibiotic alone and in combination with supernatant on multidrug-resistant pathogenic bacteria

Multiresistant bacteria	Antibiotic disc (+ supernatant)	Inhibition zone (mm)
*Escherichia coli*	Ampicillin	Absent
	Ampicillin (+ S1B)	9,78 ±0,87
	Ampicillin (+ S9BI)	21,87 ±3,04
	Penicillin G (+ S1B)	9,72 ±0,61
	Penicillin G (+ S9BI)	11,18 ±0,20
	Penicillin G (+ S45)	9,3 ±0,13
*Staphylococcus aureus*	Ampicillin	Absent
	Ampicillin (+ S9A)	15,62 ±1,28
	Ampicillin (+ S20)	16,63 ±0,73
	Ampicillin (+ S39B)	16,19 ±1,27
	Ampicillin (+ S47)	16,25 ±0,74
	Ampicillin (+ S54B)	20,74 ±0,69
*Klebsiella pneumoniae*	Penicillin G	Absent
	Vancomycin	Absent
	Penicillin G (+ S9BI)	11,84 ±0,96
	Vancomycin (+ S1B)	13,87 ±1,60
	Vancomycin (+ S9A)	14,25 ±1,24
	Vancomycin (+ S54B)	16,01 ±1,93

The resistance modulation assay showed a synergistic effect of six supernatants on multidrug-resistant *E. coli*, with halos ranging from 9.3 to 11.18 mm. We observed 3 supernatants with synergistic effect with Vancomycin and 1 with Penicillin G effective against KPC, with emphasis on Vancomycin associated with the S54B supernatant (inhibition zone of 16.01 mm), and 6 for *Staphylococcus aureus* (all with Ampicillin) and supernatant S54B also stood out in this microorganism (inhibition zone of 16.01 mm). No effect was observed for multidrug-resistant *Pseudomonas aeruginosa*.

In total, ten supernatants had a synergistic effect with antibiotics tested, some presented synergism for more than one species of multidrug-resistant bacteria, especially: supernatant S1B, effective on *E. coli* when in synergy with Ampicillin (9.78 ±0.87) and Penicillin G (9.72 ±0.61). *K. pneumoniae* when in synergy with Vancomycin (13.87 ±1.60); S9BI supernatant, effective on *E. coli* when in synergy with Ampicillin (21.87 ±3.04) and Penicillin G (11.18 ±0.20) and *K. pneumoniae* when in synergy with Penicillin G (11.84 ±0.96); S54B, effective on *S. aureus* when in synergy with Ampicillin (20.74 ±0.69) and *K. pneumoniae* when in synergy with Vancomycin (16.01 ±1.93).

## Discussion

With the advent of antibiotics, the excessive use and inadequate consumption of these drugs led to the rapid emergence of multidrug-resistant pathogens. Among these, Gram-positive bacteria, such as *Staphylococcus aureus*, and Gram-negative, such as *Escherichia coli*, *Klebsiella pneumoniae*, and *Pseudomonas aeruginosa*, stand out for their high ability to develop multiple mechanisms of antimicrobial resistance, which makes them several global public health problems [1, 26]. With this, treating a bacterial infection in modern medicine has overwhelmed researchers and pharmaceutical companies to develop new effective antimicrobials against these multidrug-resistant pathogens of arduous treatment [27, 28].

Several authors suggest that the only way to contain the current antimicrobial resistance crisis will be to develop entirely new strategies to combat these pathogens. Such as the combination of antimicrobial drugs with other agents that neutralize and obstruct the mechanisms of antibiotic resistance expressed by the pathogen [5, 6, 28–30]. In this sense, different studies have sought new bioactive substances with antimicrobial effects from microorganisms isolated from the environment. And the production of biomolecules by bacteria isolated from by-products of stingless bees represents considerable potential for bioprospecting of new compounds with antimicrobial effect [8–11, 31–34].

In the larval food of stingless bees, several species of microorganisms provide digestive enzymes, which participate in the pre-digestion of food stocks, as well as organic acids and antibiotics, which start the development of concurrent microorganisms [35, 36] Our study showed that bacteria isolated from the larval food of *Melipona quadrifasciata*, *Melipona scutellaris* and *Tetragonisca angustula* had antimicrobial activity against Gram-positive and Gram-negative bacteria, including multidrug-resistant strains. However, in general, by the antimicrobial activity assay through the PIC, it was possible to verify that the supernatants had a higher antimicrobial effect on the sensitive pathogenic bacteria when compared to their application in resistant strains. This can be explained by the fact that multidrug-resistant bacteria have several mechanisms of escape of antimicrobial molecules, including not only the production of enzymes but also the production of flow pumps and changes in membrane permeability, which prevent the accumulation of bactericidal substances inside the microbial cell [37]. The modulation assay showed that some supernatants reestablished the effect of antibiotics tested on resistant strains Gram-positive, *S. aureus* (MRSA) and Gram-negative- *E. coli* (APEC) and *K. pneumoniae* (KPC), with no effect on *P. aeruginosa*.

Many studies portray the difficulty in finding effective substances to inhibit the growth of gram-negative bacteria. Carneiro et al. [32] evaluated the antimicrobial potential of pollen extract and propolis extract of *M. compressipes manaosensis* (jupará) in *E. coli* and did not obtain significant results in the analyses performed. Similarly, Tenorio et al. [38] did not visualize the inhibiting action of *Melipona fasciculata* honey for *E. coli* and *P. aeruginosa*. In a recent investigation, Torres et al. [10] demonstrated significant inhibition of *E. coli* and *K. pneumoniae* with geopropolis extract in *Melipona quadrifasciata quadrifasciata* and *Tetragonisca angustula*, but with more effect in Gram-positive bacteria.

This study demonstrated a higher bactericidal effect of supernatants on methicillin-resistant *S. aureus* (MRSA) strains than against sensitive strains, different from that found in Gram-negative bacteria. Several studies have demonstrated a possible bactericidal action against MRSA from by-products of stingless bees or biomolecules produced by the associated microbiota [10, 32, 39–42]. Jenkins et al. [43] found that the expression of MRSA genes decreased virulence due to exposure to different concentrations to Manuka honey and that, although the antimicrobial effect has it found, the mode of inhibition of quorum sensing of these bacterial cells have not yet it found, indicating the need for further studies.

The research by Torres et al. [10] investigated the antibacterial action of the ethanol extracts of geopropolis (EEP) of *Melipona quadrifasciata quadrifasciata* and *Tetragonisca angustula.* Finding greater efficacy of the EEPs of *M. quadrifasciata quadrifasciata* against gram-positive strains than gram-negative, especially against Methicillin-resistant *S. aureus* and *S. aureus* compared to *T. angustula* extract, by a mechanism that involves disturbance of the integrity of the bacterial cell membrane. In the study by Nishio et al. [41], the antibacterial activity of honey produced by stingless bees *Scaptotrigona postica* and *Scaptotrigona bipunctata* against methicillin-resistant *Staphylococcus aureus* (MRSA) and sensitive *S. aureus* strains was verified. A recent study demonstrated broad inhibiting activity against MRSA strains by the supernatant of *Bacillus velezensis* isolated from stingless bees [44]. However, contrary to our study, most authors also report relevant antimicrobial action on Sensitive *S. aureus* (RRR). Since MRSA is a strain of *S. aureus* with a mutation of the antibiotic action site, that is, the penicillin-binding protein (PBP), which is now called PBP2a [45], we can suggest that this mutated protein has been the target of the antimicrobial action of supernatants, making the resistant microorganism more vulnerable.

And all identified bacteria are related to the intestinal tract of bees or some insects. *Serratia marcescens* and *Providencia rettgeri* were isolated from the intestine of bees, the former being recognized as an opportunistic pathogen [46]. Furthermore, metabolites produced by *S. marcescens*, such as serrawettins, have the capacity and inhibition of gram-positive and gram-negative bacteria that present an antimicrobial resistance profile [47]. *Enterococcus faecalis* has been described colonizing the surface of nests of *Melipona quadrifasciata* and the species *Alcaligenes faecalis* is a fecal coliform found in the species *Trigona spinipes* [48]. Secondary metabolites of *Vagococcus fluvials* have been described as inhibiting the growth of bacteria such as *Pseudomonas aeruginosa*, *Vibrio alginolyticus*, and *Aeromonas hydrophila* [49], as well as in our study, where they inhibited the growth of multiresistant *P. aeruginosa* and KPC. Indicating that these organisms have great potential for discovering new molecules with antibiotic activity.

In this context, necessary researches seek biomolecules that act in synergy with antimicrobials used to treat infections by Gram-negative and Gram-positive pathogens. Currently, combination therapy is a growing study strand because of its potential to reduce the resistance of bacteria to antibiotics and have fewer adverse effects [50]. The resistance modulation assay showed that some supernatants had a synergistic effect against resistant bacteria, indicating the existence of molecules that act together with the antibiotic to inhibit microbial growth. Of the antibiotics tested, ampicillin had satisfactory results against *S. aureus* MRSA when combined with six supernatants, which strengthens the hypothesis of the vulnerability of this strain to the action of the antibiotic together with antimicrobial biomolecules from the supernatant, which can act on PBP2a [51]. Gram-negative bacteria were also sensitive to the joint action of antibiotics associated with supernatants, indicating the existence of molecules responsible for binding to the penicillin-binding site in the case of resistance to penicillin and ampicillin. The vancomycin resistance is positively regulated by the VanS kinase receptor that may be interacting with antimicrobial peptides and allowing vancomycin to bind to receptors [52]. Effects were not found in *Pseudomonas aeruginosa*, which can be explained by the multiple intrinsic and plasmid resistance mechanisms that this pathogen can exhibit [53, 54].

## Conclusion

This research, all data obtained and the analyses we perform pave the way for further studies on the molecules produced by these microorganisms to be used as antibiotics alone or in synergy with antibiotics already established in the market. It has been shown here that larval food bacteria from stingless bees produce supernatants with bioactive molecules that have the potential to inhibit the growth of antibiotic-resistant microorganisms. The next step is the identification and characterization of molecules that have an antimicrobial effect.

## Data Availability

All data generated or analysed during this study are included in this published article.
